# The Johns Hopkins Hospital Reports

**Published:** 1892-12

**Authors:** 


*The Johns Hopkins Hospital Reports.* Vol. II. Nos. 7, 8, 9. Report in Pathology, I. Amœbic Dysentery. By William T. Councilman, M.D., and Henri A. Lafleur, M.D. Pp. 156. Baltimore: The Johns Hopkins Press. 1891.

The amœba hitherto has been a useful ally of the physiologist in elucidating the properties of protoplasm and in demonstrating the simplest phenomena of life; but only recently has it been shown to have an important influence in the etiology of dysentery. In the year 1859 Lambl first described amœbæ in the stools, and afterwards Cunningham found them to be abundant in cholera patients; but they were considered to be accidentally present until the year 1875, when Lösch gave in Virchow's archives an accurate description of an amœba coli which he had found in the stools of a dysenteric patient, and which he believed to be the essential cause of the disease. Amœbæ were afterwards found in the stools of dysenteric patients by various Italian authors, and then by Koch in his examination of the intestines of patients dead of dysentery in Egypt. In 1886 Kartulis, of Alexandria, endeavoured to prove that the amœbæ were the cause of tropical dysentery, and Hlava, in Prague, came to the same conclusion regarding European dysentery. Numerous observations have been recorded in support of this view, but of these by far the most important is that which emanates from the Johns Hopkins Hospital at Baltimore. This report is partly clinical, and contains an account of fourteen cases of amœbic dysentery, carefully analysed and recorded. In all of these cases amœbæ were found, either during life or after death, and their presence was found to be of diagnostic value, inasmuch as they were not found in cases of catarrhal, diphtheritic, or membranous colitis.

Six of the cases were complicated by abscess of the liver, and amœbæ were found in great numbers scattered throughout the contents of the abscesses; there were no abscesses in which the amœbæ were not found. They were more numerous in the smallest and most recent of these, and in those situations in which the abscess was extending; there were no other organisms in these small abscesses. Abscess of the lung, secondary to that of the liver, was found in three cases, and the sputum in these contained characteristic amœbæ. The authors have done much towards the investigation of dysentery, and its relation to hepatic and pulmonary abscesses. They appear to be justified in considering the following summary to rest upon a fairly well established basis:

1. Amœbic dysentery is a form of dysentery which etiologically, clinically, and anatomically should be regarded as a distinct disease.

2. The amœba dysenteriæ has been shown to be the causative agent, from its constant presence in the stools and in the anatomical lesions, and from the inoculation experiments by Kartulis.

3. Clinically the disease is characterised by the presence of amœbæ in the stools, which, in addition, present physical characters different from those seen in the stools of other forms of dysentery.

4. Anatomically the disease is characterised by the production of ulcers in the colon, which generally differ from those found in any other forms of dysentery.

5. Abscess of the liver, with or without involvement of the lungs, is a frequent complication, much more so than in any form of dysentery.

6. The disease is widely distributed, and is found in most countries in Europe, in most parts of the United States, and in the tropics everywhere.

7. This is the form of dysentery commonly called tropical.

### Notes on Preparations for the Sick.

